# Disease Germs and the Telephone

**Published:** 1899-04

**Authors:** 


### DISEASE GERMS AND THE TELEPHONE.

THAT disease germs are contagious in very many ways is a well-known fact. It is now recognized as a great risk for two people to live together in the same room if one should be afflicted with pulmonary tuberculosis. We have recently spoken of contagion occurring in barber-shops, and we might also mention in Pullman cars. Now comes the fact recently discovered that disease germs may be contracted at the telephone, not over the wires, but by coming in contact with the transmitters in talking and listening. Here is what the *Brooklyn Medical Journal* has to say:

"Much alarm has been excited by the reported discovery that the telephone acts not only as a transmitter of sound, but of contagion as well. We are told by the daily press that an expert chemist in New York has found in the transmitters which he has examined the germs of consumption, smallpox, scarlet fever, the grip, besides other bacteria too numerous to mention. This is certainly a startling discovery, and adds another to the claims already made for the telephone, inasmuch as up to the present time bacteriologists have sought in vain by all means known to them for the germs of smallpox and scarlet fever, and yet for years they have been under their very nose, as it were, awaiting the keen eyes of the expert chemist. That telephone transmitters may become and often are filthy we have no doubt, and a proper and systematic cleansing is not only desirable but imperative, but to accomplish this we think it is hardly necessary to alarm the world at large, and announce discoveries which are so ridiculous as to cause every one at all familiar with the subject to smile aloud."

---

ONE of our Western exchanges has published an article by a physician which shows the most remarkable originality that it has ever been our good fortune to see. His subject is on the vaginal irrigator, and for fear that our own language may be too weak to express the terse description of this Western physician we will quote him *verbatim*, for it is seldom that a physician shows such familiarity with a subject as is seen in this article. He says: "To obviate these troubles (*i. e.*, badly constructed vaginal irrigators) I sought another form, and in so doing the idea occurred to me that Nature had never intended that syringe nozzles, irrigators or any other instrument made by hand of man should enter the vagina. There is only one instrument which was created for that purpose, and that was fashioned by the Great Creator, who saw fit to make this instrument the same shape in all of the mammalia. It is the general experience of the world that this instrument can easily enter the vagina, no matter what the position of the parties; it can be used in the sitting posture, standing up, lying on the back or face, in the genupectoral position, or when lying on either side. For general all-round adaptability it seems unexcelled, and from the time of Adam up to date, no one has seen fit to improve thereon." He then goes on to tell that he devised a nozzle irrigator in the shape of a penis, and that success was then assured. Surely "there is nothing new under the sun," and this unique invention may in the future revolutionize the work of the gynecologist.

---

According to the *British Medical Journal,* Dr. Menahem Hodarn, of Constantinople, has brought forth the new experimental idea of curing baldness of the head surgically. It seems as if the doctor had two cases of favus under treament, and not being satisfied with simply curing the disease he undertook to cure the baldness resulting therefrom. According to this journal his plan was to scarify the bare surface and to plant thereon hairs removed from other parts of the patient's head. The hairs used for this purpose were trimmed with scissors at each end. Some four weeks after implantation a certain number of the hairs were found to have taken root, and in no long time a goodly new crop was produced. Encouraged by these results Dr. Hodarn has since applied the method in other cases of baldness following favus, and he thinks himself justifiable in stating that "clinically there can be no doubt as to this very curious fact—that small bundles of hair stems cut with scissors and implanted in the incisions made with the scarifier can take root and grow, forming in time long and viable hairs. By microscopic examination he has satisfied himself that a real bulb forms at the lower end of the hair after some weeks." The *Journal* asks, "Why should not the same treatment be applied in cases of ordinary baldness?" Certainly this experiment opens up a wider field for investigation.

THE evil effects of tight lacing are so universally recognized by medical men, that it is no surprise to us that laws should be contemplated to try to regulate such an evil. In St. Petersburg, according to the *British Medical Journal*, a ministerial decree has been issued to the effect that the girls attending universities, schools of music and fine arts, and other educational establishments of the same order, must on entering "doff the cuirass which they wear under the name of corset." Recently Mr. Dagget, of Wisconsin, has introduced in his legislature a bill to this effect: "Resolved, That a committee be appointed, consisting of three members, to draft a bill to protect the health of the misses, old maids and married women of the State of Wisconsin, by making a law to prohibit tight-lacing." We are thorough advocates of personal liberty, and we cannot subscribe, therefore, to such a State law. Every family ought to have its family physician to whom they can go for advice, and if he should tell the women of the household that tight-lacing is injurious, then that is enough, and no law should be passed which savors of paternalism. If a woman thinks she looks more beautiful by being laced tight, then why should there be a law to dispel such illusions in her mind? It is better to be thought beautiful than not to be beautiful at all.

---

A GREAT change has come over the profession in the last few years in regard to wounds of the heart. Slight wounds of this organ were formerly considered quite unfavorable, and in the question of shooting to kill, it was always thought best to aim right at the heart. Now it is known that such is not the most vital part of the body, for only recently in New York the coroner found a bullet in the pericardial sac which had passed through a good portion of the heart substance, and yet the patient had lived for fourteen days after receiving the wound. Dr. Burtenshaw, in the *Medical News* for March 11th, reviewed the results of more than 100 cases of paracentesis of the pericardium, among which number there was only one serious result. He himself also reports an interesting case occurring in his own practice. Laforgue has given the statistics of 56 cases substantiated by autopsy which is exceedingly interesting. In 56 cases of wounds of the heart 18 were followed by immediate death, 21 patients survived for a longer or shorter period, and 17 completely recovered. Surgery of the heart therefore opens up a broad field for future developments.

---

IN the last issue of THE JOURNAL we mentioned the case of a child which had bled to death from the extraction of a tooth. This was a case of hemophilia, the patient usually being designated as a "bleeder." While these cases are rare they do sometimes occur, and it has long been a problem of how to deal with such cases in the *interim* so as to put the system in a condition antagonistic to the bleeding tendency. Various remedies have been used, seemingly without success. In the *British Medical Journal* of March 4, Dr. Siddall calls attention to a remedy which he has used with marked success in three cases, a fact which justifies him in bringing it to the attention of the profession. The remedy is the administration internally to an adult, "20 minims of dilute sulphuric acid in an ounce of water three times a day." The remedy is certainly worthy of the trial.

---

IT is with a feeling of sadness that we must chronicle the death of one of Atlanta's oldest physicians and surgeons, Dr. Kinsman C. Divine. Dr. Divine was stricken suddenly with death on the afternoon of March 21, while engaged in the performance of a surgical operation. The cause was evidently that of angina pectoris, as a few moments before he fell he complained of intense pain in the region of the heart. Dr. Divine was a most active member of the Confederate Veterans, holding the position of surgeon, and was always prominent in all celebrations of this organization. He was an active member of the Georgia Medical Association and the Atlanta Society of Medicine. In the last few years of his practice Dr. Divine had limited his practice largely to rectal diseases.

---

THE daily papers have announced to us the death of "Cavendish," the celebrated authority on whist and kindred games. According to the *British Medical Journal* it was not well known that "Cavendish" was a medical man, and that his real name was Henry Jones. This journal tells us that Mr. Jones was the eldest son of a well-known practitioner in Saho Square, and was himself a member of the Royal College of Surgeons of England and a Licentiate of the Royal College of Apothecaries. He died on Feb. 10, at the age of 68.

---

DR. GEORGE H. SIMMONS, of Lincoln, Nebraska, has been elected by the board of trustees as editor of the *Journal of the American Medical Association,* to succeed the late Dr. J. B. Hamilton. He will assume control about March 1st, and is to devote his entire time to the interests of the *Journal,* the salary of the position having been advanced from $4,000 to $5,000. Dr. Simmons has been for a number of years the able editor of the *Western Medical Review,* published at Lincoln, and besides being a versatile writer, has executive ability of a marked order. He received the degree of M.D. at Rush Medical College in 1892, having ten years before, however, taken a degree from the Hahnemann Medical College and Hospital, of Chicago, and has been an active member of the A. M. Association since 1897.

